# Cyclooxygenases and prostaglandin E_2 _receptors in growth plate chondrocytes *in vitro *and *in situ *– prostaglandin E_2 _dependent proliferation of growth plate chondrocytes

**DOI:** 10.1186/ar1948

**Published:** 2006-04-28

**Authors:** Christoph Brochhausen, Pia Neuland, C James Kirkpatrick, Rolf M Nüsing, Günter Klaus

**Affiliations:** 1 Institute of Pathology, Johannes Gutenberg-University, Mainz, Germany; 2 Department of Pediatrics, Philipps-University, Marburg, Germany; 3 Institute of Clinical Pharmacology, Johann Wolfgang Goethe-University, Frankfurt/Main, Germany

## Abstract

Prostaglandin E_2 _(PGE_2_) plays an important role in bone development and metabolism. To interfere therapeutically in the PGE_2 _pathway, however, knowledge about the involved enzymes (cyclooxygenases) and receptors (PGE_2 _receptors) is essential. We therefore examined the production of PGE_2 _in cultured growth plate chondrocytes *in vitro *and the effects of exogenously added PGE_2 _on cell proliferation. Furthermore, we analysed the expression and spatial distribution of cyclooxygenase (COX)-1 and COX-2 and PGE_2 _receptor types EP1, EP2, EP3 and EP4 in the growth plate *in situ *and *in vitro*. PGE_2 _synthesis was determined by mass spectrometry, cell proliferation by DNA [^3^H]-thymidine incorporation, mRNA expression of cyclooxygenases and EP receptors by RT-PCR on cultured cells and in homogenized growth plates. To determine cellular expression, frozen sections of rat tibial growth plate and primary chondrocyte cultures were stained using immunohistochemistry with polyclonal antibodies directed towards COX-1, COX-2, EP1, EP2, EP3, and EP4. Cultured growth plate chondrocytes transiently secreted PGE_2 _into the culture medium. Although both enzymes were expressed in chondrocytes *in vitro *and *in vivo*, it appears that mainly COX-2 contributed to PGE_2_-dependent proliferation. Exogenously added PGE_2 _stimulated DNA synthesis in a dose-dependent fashion and gave a bell-shaped curve with a maximum at 10^-8 ^M. The EP1/EP3 specific agonist sulprostone and the EP1-selective agonist ONO-D1-004 increased DNA synthesis. The effect of PGE_2 _was suppressed by ONO-8711. The expression of EP1, EP2, EP3, and EP4 receptors *in situ *and *in vitro *was observed; EP2 was homogenously expressed in all zones of the growth plate *in situ*, whereas EP1 expression was inhomogenous, with spared cells in the reserve zone. In cultured cells these four receptors were expressed in a subset of cells only. The most intense staining for the EP1 receptor was found in polygonal cells surrounded by matrix. Expression of receptor protein for EP3 and EP4 was observed also in rat growth plates. In cultured chrondrocytes, however, only weak expression of EP3 and EP4 receptor was detected. We suggest that in growth plate chondrocytes, COX-2 is responsible for PGE_2 _release, which stimulates cell proliferation via the EP1 receptor.

## Introduction

Prostaglandins, especially prostaglandin E_2 _(PGE_2_), play an important role in bone and cartilage metabolism. Although PGE_2 _was initially described as a potent bone-resorbing substance [1], several studies have demonstrated its activity in bone-forming processes [2,3]. In osteoblast-like cells, endogenous PGE_2 _was shown to affect proliferation and differentiation by stimulation of DNA synthesis and alkaline phosphatase activity [4]. An interesting aspect in the investigation of the function of prostaglandins in cartilage or bone tissue is their possible role in the growth plate. This special cartilage tissue is responsible for the endochondral ossification of long bones and represents all differentiation steps in distinguishable layers, from undifferentiated reserve zone cells to proliferative and hypertrophic chondrocytes, which initiate cartilage mineralisation. Due to this complex structure of the growth plate, cellular effects of prostaglandins on growth plate chondrocytes have been examined using various *in vitro *systems. PGE_2 _elicits differentiation of chondrocytes, as previously shown for the chondrocyte cell line RCJ3.1C5.18 [5] and rat growth plate chondrocytes [6]. In the latter, the effect of PGE_2 _was mediated by cAMP and protein kinase C. Furthermore, PGE_2 _also makes an important contribution to cartilage formation and promotes DNA and matrix synthesis in growth plate chondrocytes [7]. In addition to various findings *in vitro*, the physiological role of prostaglandins was clarified by its stimulating effect on bone formation and by the increase in bone mass after systemic administration of PGE_2 _to infants [8] and animals [9]. Furthermore, local administration of PGE_2 _resulted in osteogenesis *in situ *[10,11].

The rate-limiting step for the synthesis of PGE_2 _and other prostaglandins is the conversion of arachidonic acid to prostaglandin endoperoxide by cyclooxygenase (COX), which exists in two isoforms, COX-1 and COX-2 [12]. These enzymes are differentially regulated. Previous *in vitro *analysis demonstrated the functional importance of COX-1 for proliferation, differentiation and matrix production in cultured growth zone chondrocytes [13]. In various chondrocyte cell models, as well as in fracture callus formation, COX-2 may also be important for prostaglandin synthesis [14]. Moreover, the expression of COX-2 is regulated by different stimuli, such as tumour necrosis factor-α [15] or shear stress [16]. The induction of COX-2 is regarded as an important step in inflammatory situations. COX-1 and COX-2 are expressed in inflamed bone tissue [17] and COX inhibitors are extensively used in the treatment of rheumatoid arthritis. However, inadequate information is available on *in situ *expression of both COX-1 and COX-2 within the growth plate to correlate *in vitro *findings with the *in situ *situation.

PGE_2_, the principal product of bone prostaglandin synthesis, acts locally on target cells by binding to prostaglandin E (EP)-type G protein-coupled receptors. Four different EP receptors are known, which are linked to different intracellular signal transduction pathways [18]. The EP1 receptor is coupled to intracellular Ca^2+ ^mobilization, while the EP2 and EP4 receptors increase intracellular cAMP accumulation. By contrast, EP3 inhibits intracellular cAMP accumulation. Regarding bone formation and bone resorption, the EP4 receptor has been shown to be essential in terms of PGE_2 _action in bone [19]. Recently, the EP2 and EP4 receptors were shown to be required for PGE_2_-dependent chondrocyte differentiation [20]. In previous studies, we demonstrated that stimulation of growth plate chondrocyte proliferation by both calciotropic hormones, 1,25 (OH)_2_D_3 _and parathyroid hormone, is dependent on an increase in intracellular calcium and activation of protein kinase C [21]. On the other hand, an increase in intracellular cAMP concentration was without any effect on proliferation [21], but was able to stimulate matrix synthesis [22]. In the present study, we were interested in whether PGE_2 _acts in a proliferative and stimulatory fashion on growth plate chondrocyte function. We therefore investigated the effects of PGE_2 _and prostaglandin receptor agonists and antagonists on cultured growth plate chondrocytes. Furthermore, we analysed the expression and spatial distribution of COX-1 and COX-2 and the PGE_2 _receptors EP1, EP2, EP3, and EP4 in the growth plate and compared this profile with their expression in cultured growth plate chondrocytes in order to give innovative insights into *in situ *-*in vitro *correlations.

## Materials and methods

### Materials

Polyclonal rabbit antibodies against the EP1, EP2, EP3 and EP4 receptors and COX-1 and COX-2 were described previously [23,24]. Polyclonal rabbit antibodies against collagen (Col) type I and type II were purchased from Biotrend Chemicals GmbH (Cologne, Germany). Monoclonal anti-collagen type X antibody (mouse) was from Quartett (Berlin, Germany). All other antibodies used were obtained from DAKO (Glostrup, Denmark). DNase (10 U/μl) for cartilage digestion was from Amersham Pharmacia Biotech (Piscataway, NY, USA) and CaCl_2 _was from Serva (Heidelberg, Germany). FCS and culture dishes were from Greiner (Frickenhausen, Germany), and culture media were obtained from PAA GmbH (Linz, Austria). Butaprost, misoprostole, sulprostone and PGE_2 _were purchased from Cayman Chemical Company (Ann Arbor, Michigan, USA). Ligands for the PGE_2 _receptors (ONO D1-004, ONO AE1-259-001, ONO AE-248, ONO AE1-329, and ONO-8711) have been described previously [25-27] and were kindly provided by Dr Maruyama (ONO Pharmaceuticals, Osaka, Japan). PicoGreen for double-stranded (ds)DNA quantification was obtained from Mobitec (Göttingen, Germany). Gene Amp RNA-PCR kit, DNA Polymerase (Ampli taq Gold), reverse transcriptase (MuLV RT) and oligo d(T)_16 _were purchased from Perkin Elmer, Roche Molecular Systems Inc. (Branchburg, NJ, USA). Other chemicals were of p.a. grade and purchased from Merck (Darmstadt, Germany), Gibco BRL Life Technologies (Karlsruhe, Germany) or Sigma Aldrich Chemistry (Steinheim, Germany).

### Cell culture

#### Isolation of chondrocytes

Chondrocytes were isolated and cultured as described earlier by Benya and Shaffer [28] and modified according to Klaus and colleagues [21]. Briefly, femurs of up to four week old Sprague Dawley rats (60 to 80 g each) were dissected. The epiphyseal growth plate of the tibiae was separated by cleaning the cartilage plate of muscular tissue, periosteum and perichondrium. The proximal epiphysis was divided by a transverse cut with a sharp scalpel, and the cartilage plate was separated distally from the calcification zone of the tibial metaphysis. Isolated growth plates were digested for 3 hours at 37°C by collagenase (0.12% w/v) and DNase (0.02% w/v) in 5 ml of serum free F12/DMEM medium. After thorough washing, cells were counted using a Neubauer chamber. Viability, examined by trypan blue exclusion, was > 95%.

#### Monolayer cultures

Chondrocytes were cultured in flasks, 96-well-plates or 2-well cell-tissue-chambers containing F12/DMEM 1/1 medium supplemented with 10% FCS, 10 mM HEPES, 2 mM pyruvate, 2 mM L-glutamine, 0.7 μM CaCl_2_, 10 mg/ml penicillin/streptomycin and L-cysteine. Ionized calcium measured by a calcium-sensitive electrode was 1.2 mmol/l. During the first four days of cell culturing the serum substitute Ultroser-G (1%) was added to the medium. From day 5 on, β-glycerophosphate (10 mM) and L-thyroxine (100 μg/μl), as well as ascorbic acid (5 to 60 μg/ml) from day 11 on, were added to the culture medium. Medium was changed every 48 hours and cells became confluent within 6 to 12 days.

### Assay of cell proliferation: semiquantitative dsDNA determination

Primary cultures of chondrocytes were transferred to 96-well-plates in serum-free medium without L-thyroxine, which is reported to exert antiproliferative effects [29]. Cell cycles were synchronised for 24 hours as described earlier [21].

PGE_2_, EP receptor agonists, or vehicle were added with fresh medium, supplemented with 10% FCS and cells were stimulated for 24 or 48 hours. Incubation was stopped by aspiration of the supernatants and the culture plates were frozen at -80°C for 1 hour. Thereafter, cells were thawed and incubated with 200 μl staining solution (containing 2.5 μl/ml PicoGreen) for 10 minutes under light protection. Optical density was determined using a plate reader (excitation/emission, 485 nm/530 nm). Experiments were run with four to six parallel aliquots.

### Assay of cell proliferation: [^3^H]-thymidine incorporation

Incorporation of [^3^H]-thymidine was determined in serum-free cultures as described previously [21]. Cells were synchronised in serum-free medium for 24 hours. Thereafter, medium was changed to F-12/DMEM with 0.2% (w/v) bovine serum albumin and the substances or vehicles were added. Cells were incubated for 48 hours and 2 μCi [^3^H]-thymidine were added to each well 3 hours before stopping the incubation.

### Reverse transcriptase-polymerase chain reaction

Total RNA was isolated from first passage monolayer cultures of chondrocytes and from two to eight freshly isolated epiphyseal growth plates that were pulverised in liquid nitrogen. After DNase digestion, 1.2 μg (from cells) or 0.5 μg (from tissue) RNA was transcribed into cDNA using oligo dT. RT-PCR was performed for EP1, EP2, EP3, EP4, COX-1, COX-2, Col I, Col II, Col X and β-actin. Primers used in this study are listed in Table 1. The amplification profile consisted of denaturation at 95°C for 30 seconds, annealing at 54°C (EP receptors and COX) or at 57°C (collagens) for 45 seconds and extension of DNA at 72°C for 30 seconds after a 10 minute denaturation step at 95°C. When using RNA from bone tissue, the number of cycles were 40 for the EP receptors and 45 for the collagen types, and when using RNA from cultured chondrocytes, 35 cycles and 30 cycles, respectively, were performed. The amplification products of 10 μl of each PCR reaction were separated on a 1.8% agarose gel, stained with ethidium bromide, and visualised by ultraviolet irradiation. Identification of amplification products was determined by size and dideoxy sequencing.

**Table 1:** Primers used for RT-PCR

mRNA	Sequence of primer	Product (bp)	Accession numbers
EP-1	5' -GCT GTA CGC CTC GCA TCG TGG-3' 5' -GTG TTT CGA GCA TCC CAT GTA TCT-3'	404	NCBI:D16338
EP-2	5' -GAA CGC TAC CTC TCC ATC GG-3' 5' -TGA TGG TCA TAA TGG-3'	415	NCBI:D50589
COX-1EP-3	5' -GTTTGGTCTG GCGTCTTAGA AC-3' 5' -CTTGGAACAG GACCTTCTGA GT-3' 5' -TTTGCCTCCGCCTTCGCCTG-3' 5' -AGCAGCAGATAAACC-3'	399,359	U03388 NCBI:D14869
COX-2EP-4	5' -AATGAGTACC GCAAA-3' 5' -ATCTAGTCTG GAGCGGGAGG-3' 5' -TGCTCATCTGCTCCATTCCGC-3' 5' -ATGCGAACCTGGAAG-5'	420,407	NM011198NCBI:D28860
Col ICOX-1	5' -TGGTGACAAG GGTGAGACAG-3' 5' -TGAGGCAGGA AGCTGAAGTC-3' 5' -GTTTGGTCTG GCGTCTTAGA AC-3' 5' -CTTGGAACAG GACCTTCTGA GT-3'	329,399	Z78279NCBI:U03388
Col IICOX-2	5' -CTCCAGGTGT GAAGGGTGAG-3' 5' -GAACCTTGAG CACCTTCAGG-3' 5' -AATGAGTACC GCAAA-3' 5' -ATCTAGTCTG GAGCGGGAGG-3'	261,420	NM012929NCBI:NM011198
Col XI	5' -TGCCTCTTGT CAGTGCTAAC C-3' 5' -GCGTGCCGTT CTTATACAGG-3' 5' -TGGTGACAAG GGTGAGACAG-3' 5' -TGAGGCAGGA AGCTGAAGTC-3'	248 329	AJ131848NCBI:Z78279
β-actinCol II	5' -CATCACCATT GGCAATGAGC G-3' 5' -CTAGAAGCAT TTGCGGTCGG AC-3' 5' -CTCCAGGTGT GAAGGGTGAG-3' 5' -GAACCTTGAG CACCTTCAGG-3'	403,261	NM031144NCBI:NM012929
Col X	5' -TGCCTCTTGT CAGTGCTAAC C-3' 5' -GCGTGCCGTT CTTATACAGG-3'	248	NCBI:AJ131848
β-actin	5' -CATCACCATT GGCAATGAGC G-3' 5' -CTAGAAGCAT TTGCGGTCGG AC-3'	403	NCBI:NM031144

Col, collagen; COX, cyclooxygenase; EP, prostaglandin E receptor.

### Immunohistochemistry

For immunohistochemistry, the epiphyseal plate with neighbouring bony metaphysis and epiphysis including the knee joint were dissected. The isolated tissue was immediately frozen in isopentane at -80°C. For detection of EP1, EP2, EP3, EP4, COX-1, COX-2, Col II and Col X, the alkaline-phosphatase-anti-alkaline-phosphatase method was used according to Cordell and colleagues [30] as modified by Bittinger and colleagues [31]. Frozen sections (4 μm) were fixed in paraformaldehyde (4%). Polyclonal rabbit antibodies against EP1 (1:300), EP2 (1:200), EP3 (1:300), EP4 (1:300), COX-1 (1:100), COX-2 (1:100) and Col II (1:800) as well as a monoclonal mouse antibody against Col X (1:200) were incubated for 16 hours at 4°C. After staining, these sections were counter-stained with hemalaun. For the antibodies directed against the EP receptors, the following controls were performed. Firstly, the primary antibody was omitted; under this condition no staining was visible. Secondly, the antibodies were preabsorbed with the corresponding peptide against which they are directed as described previously [24]; under this condition staining was completely blocked.

### Determination of PGE_2_

PGE_2 _was determined in cell supernatants as described previously [32].

### Statistical analysis

Statistical analysis was carried out by *t *test or ANOVA as appropriate. *P *values are < 0.05 or < 0.001.

## Results

### Collagen expression in cultured chondrocytes

**Figure 1:**
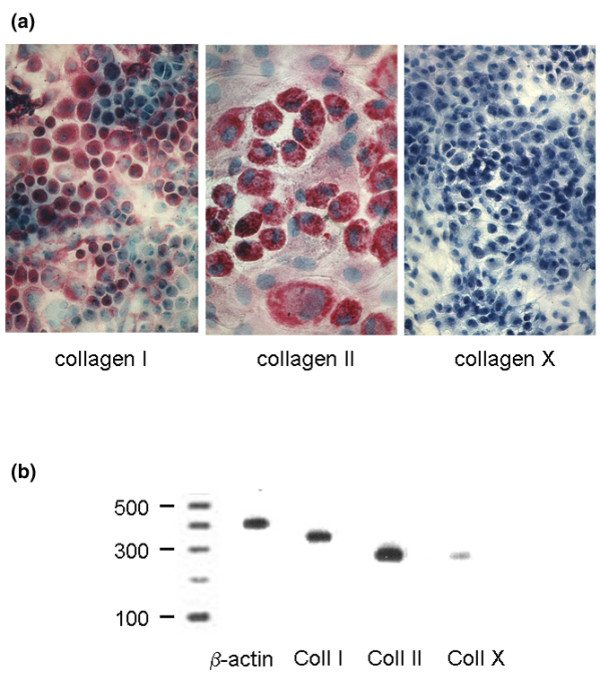
Collagen protein and mRNA expression in cultured rat growth plate chondrocytes. Isolated rat chondrocytes were cultured until confluency. **(a) **Protein expression for collagen I, II and X was studied in cultured chondrocytes with type-specific antibodies and using the alkaline-phosphatase-anti-alkaline-phosphatase method. Collagen type I was expressed in the majority of the cultured cells. Collagen II was strongly detected in chondrocytes of polygonal shape, representing more than 80% of the cultured cells. In cultured chondrocytes, no reactivity towards the collagen X antibody was observed. The antigens of the antibodies are indicated below the figures. **(b) **mRNA expression of the various collagen types. PCR analysis revealed expression of mRNA for collagen (Coll) I and collagen II and only marginal expression of collagen X mRNA.

To define the differentiation stage of cultured chondrocytes we first studied the expression of various collagens. Col I is typically expressed towards the metaphyseal zone, whereas Col II is present in the proliferation zone and Col X in the hypertrophic zone. Proliferating cells express Col II and Col X is strongly expressed after the transition from pre-hypertrophic, proliferating chondrocytes to hypertrophy. Accordingly, we observed staining for Col II mainly in the proliferative zone and Col X in the hypertrophic zone of the growth plate (data not shown). In cultured chondrocytes, we observed strong staining for Col II in more than 90% of the cells but no antigenicity towards the anti-Col X antibody (Figure 1a). In addition, Col I was expressed in cultured chondrocytes. In support of this observation, we obtained strong amplification with specific primers for Col I and Col II, but weak amplification with oligonucleotides specific for Col X in the cultured chondrocytes (Figure 1b). This finding is in keeping with the chondrocyte phenotype, as most cells are in the proliferative stage.

### PGE_2 _production and COX-1 and COX-2 expression

Isolated rat growth plate chondrocytes released PGE_2 _transiently into the supernatants. Within the first 48 hours, a four-fold increase in PGE_2 _concentration was observed (Table 2). After six days of culture, however, PGE_2 _release by subconfluent, slowly proliferating cells was reduced almost to baseline levels.

**Table 2:** Release of PGE_2 _into the supernatant of cultured rat chondrocytes

Incubation time	PGE_2 _(μg/ml)	Proliferation status
0	120 ± 20	
2 days	530 ± 270^a^	Rapidly proliferating
6 days	150 ± 30	Slowly proliferating

Chondrocytes were seeded in culture plates and fresh medium was added. At different time points supernatant was collected and analyzed for prostaglandin E_2 _(PGE_2_) content (*n *= 6; ^a^*p *< 0.01 versus day 0).

**Figure 2:**
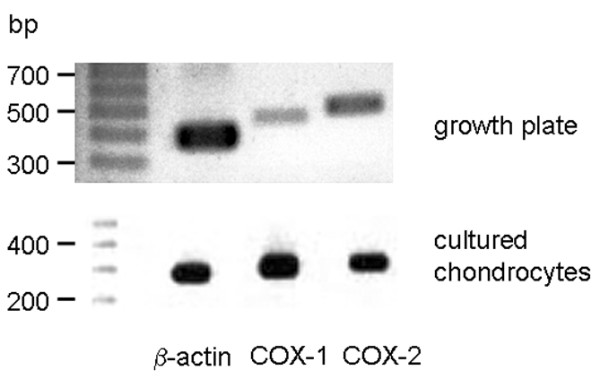
Cyclooxygenase (COX) expression in cultured rat growth plate chondrocytes and in the growth plate. Expression of mRNA for COX-1 and COX-2 was analysed by reverse transcription RT-PCR. β-actin was used as positive control. Both growth plate tissue and cultured chondrocytes express mRNA for COX-1 and COX-2. bp, base-pairs.

**Figure 3:**
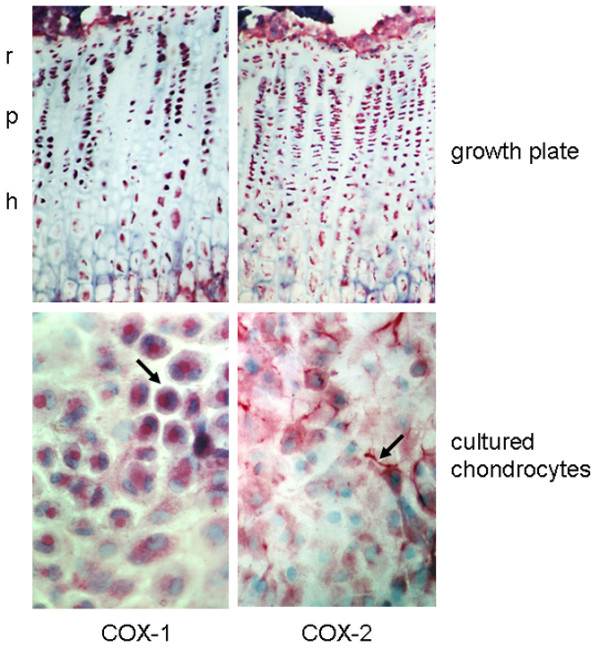
Cyclooxygenase (COX) expression in rat growth plate chondrocytes *in vitro *and *in situ*. Protein expression of COX-1 and COX-2 was studied using isoform-specific antibodies. Both COX isoforms could be detected in all zones of the growth plate. In cultured growth plate chondrocytes, COX-1 was expressed in all cultured chondrocytes with high intensity in paranuclear areas (marked by arrow). COX-2 protein was detected in extranuclear regions as well as in cell processes (marked by arrow) of a sub-population of the cultured cells only. r, reserve zone; p, proliferative zone; h, hypertrophic zone.

**Figure 4:**
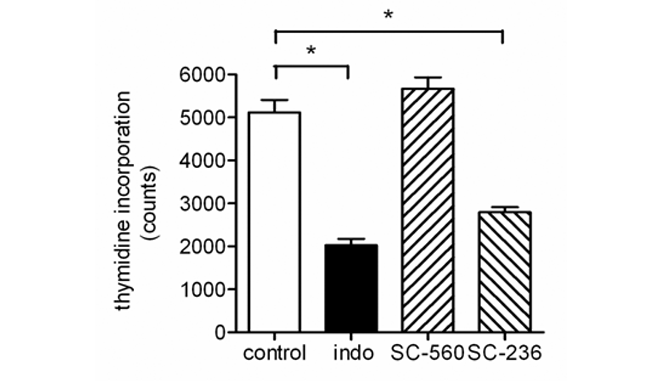
Proliferation assay with selective and unselective cyclooxygenase (COX) inhibitors. The effect of selective and unselective COX inhibitors on chondrocyte proliferation was assessed by [^3^H]-thymidine incorporation. Subconfluent chondrocytes were synchronized in serum-free medium for 24 hours. Medium was renewed and the indicated inhibitors were added for 24 hours: indo, 50 μM indomethacin; SC-560, 10 μM; SC-236, 10 μM. Data are given as mean ± standard error of the mean, *n *= 6; *p value < 0.05.

To determine the COX isoform involved in PGE_2 _synthesis, we analysed mRNA and protein expression of COX-1 and COX-2 in growth plates as well as in cultured chondrocyte*s*. Regarding mRNA expression, both growth plates and cultured chondrocytes expressed COX-1 and COX-2 mRNA (Figure 2). Isoform-specific antibodies were used to determine COX distribution in rat growth plate tissue and in cultured rat chondrocytes. To ensure specificity, the following control experiments were performed: firstly, the primary antibody was omitted; and secondly, for COX-2, the antibodies were preabsorbed with the corresponding peptide against which they are directed, as described previously [24]. Under these conditions, no staining was visible (data not shown). On the protein level, growth plates as well as cultured chondrocytes expressed both COX isoforms (Figure 3). Growth plate chondrocytes *in situ *showed intracellular expression of both COX isoforms. Regarding the spatial distribution of COX expression in the different zones of the growth plate, a disparate expression pattern of COX-1 and COX-2 was observed. COX-1 stained chondrocytes in all zones of the growth plate strongly and homogenously, whereas COX-2 appeared to be only moderately expressed in the reserve zone cells but strongly expressed in the other zones of the growth plate. In cultured chondrocytes, COX-1 expression appeared to be predominantly in the perinuclear region, whereas COX-2 expression dominated in the dendritic processes of all cells. To further investigate the role of the COX isoform in chondrocyte proliferation, we blocked both isoform activities with the unspecific inhibitor indomethacin and each of the isoforms with the specific COX-1 inhibitorSC-560, or the COX-2 inhibitor SC-236. Indomethacin suppressed chondrocyte proliferation as assessed by thymidine incorporation (Figure 4). A similar extent of proliferation inhibition was achieved by the addition of the COX-2 inhibitor SC-236 but not SC-560. This indicates that COX-2 is primarily important for chondrocyte proliferation.

### Effect of PGE_2 _and analogues on proliferation of growth plate chondrocytes

**Figure 5:**
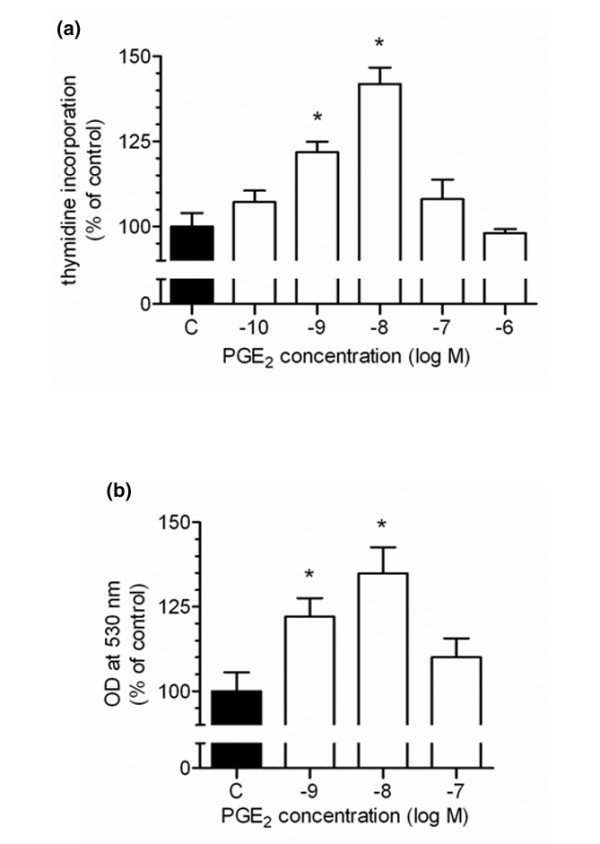
Effect of prostaglandin E_2 _(PGE_2_) on chondrocyte proliferation. **(a) **Proliferation of cultured chondrocytes was determined by [^3^H]-thymidine incorporation. Subconfluent chondrocytes were synchronized in serum-free medium for 24 hours. Medium was renewed and PGE_2 _or solvent was added in the indicated concentrations for 24 hours. Data are presented as mean ± standard error of the mean, *n *= 5. **(b) **Relative quantification of DNA in cultured chondrocytes was used as a measure for proliferation. Chondrocytes were grown in 96-well-plates until subconfluency. After synchronization, PGE_2 _or solvent was added for 24 hours. Thereafter, medium was aspirated, DNA was extracted by freeze-thawing and 200 μl of the staining solution (containing a fluorescent nucleic acid stain) were added and DNA-bound fluorophore was determined by fluorescence spectroscopy, expressed as OD at 530 nm. Data are presented as mean ± standard error of the mean of four parallel experiments, given as percent of the control. Excitation of the control was 14,705 ± 2,675 after 24 hours. *p value < 0.05.

To analyse whether PGE_2 _might stimulate cell proliferation in an autocrine or paracrine manner, we studied the effect of exogenously added PGE_2 _in cultured rat chondrocytes. Cell cycles were synchronized by 24 hour starving. DNA synthesis was determined by [^3^H]-thymidine incorporation and DNA content by fluorescence spectroscopy. In a bell-shape manner, PGE_2 _stimulated DNA synthesis with a maximum at 10 nM PGE_2 _(Figure 5a). The proliferative effect of PGE_2 _was also observed by semiquantitative determination of DNA content (Figure 5b).

**Figure 6:**
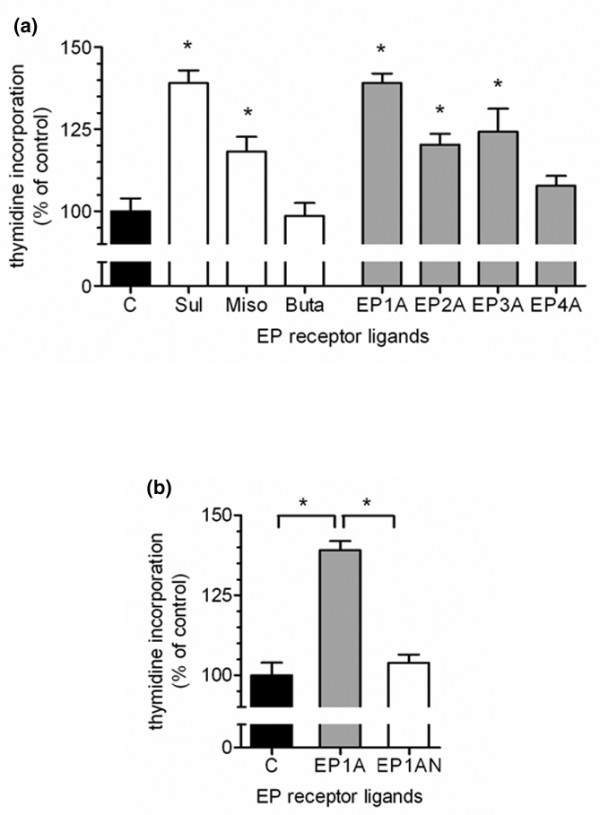
Effect of prostaglandin E (EP) receptor ligands on proliferation of cultured chondrocytes. **(a) **Unselective and selective EP receptor agonists were administered to cultured chondrocytes. Subconfluent chondrocytes were synchronized in serum-free medium for 24 hours and EP receptor agonists were added for 24 hours. Proliferation was assessed by [^3^H]thymidine incorporation. C, control; Sul, 1 μM sulprostone; Miso, 1 μM misoprostole; But, 1 μM butaprost; EP1A, 4 μM ONO-D1-004; EP2A, 0.1 μM ONO-AE1-259-01; EP3A, 0.1 μM ONO-AE-248; EP4A, 0.1 μM ONO-AE1-329. Data are given as mean ± standard error of the mean, *n *= 5. *P value < 0.05. **(b) **To study EP1 function for cell growth, a EP1 receptor selective agonist and antagonist were added to cultured chondrocytes. Subconfluent chondrocytes were synchronized in serum-free medium for 24 hours and EP1 receptor agonist (EP1A) or antagonist (EP1AN) combined with 10 nM prostaglandin E_2 _were added for 24 hours in the presence of [^3^H]-thymidine. EP1A, 4 μM ONO-D1-004; EP1AN, 1 μM ONO-8711. Data are given as mean ± standard error of the mean, *n *= 5. *P value < 0.05.

To define the EP receptor(s) involved in PGE_2 _signalling in this experimental setting, we used agonists for the various EP receptor types. Stimulation with the EP1/EP3 receptor agonist sulprostone resulted in a significant increase of chondrocyte [^3^H]-thymidine incorporation, whereas the EP2/EP3 receptor agonist misoprostole had an intermediate effect and the EP2 agonist butaprost exerted no effect (Figure 6a). These observations were further supported by the use of EP receptor subtype-specific ligands. The EP1 agonist ONO-D1-004, and to a lesser extent the EP2 agonist ONO-AE1-259-01 and the EP3 agonist ONO-AE-248, significantly increased [^3^H]-thymidine incorporation whereas the EP4 selective agonist ONO-AE1-329 exerted no effect. The proliferative activity of the EP1 agonist ONO-D1-004 was similar to maximal stimulation achieved by PGE_2_. In support of this observation, the addition of the selective EP1 antagonist ONO-8713 completely blocked PGE_2_-induced proliferation (Figure 6b).

### Expression of EP1 and EP2 receptors

**Figure 7:**
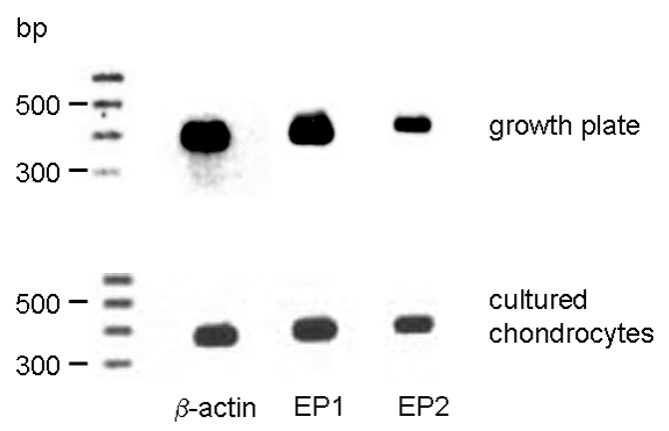
Expression of EP1 and EP2 receptors in rat growth plates and in cultured chondrocytes at the mRNA level. Expression of mRNA for EP1 and EP2 receptors was analysed by reverse transcription RT-PCR. β-actin was used as a positive control. Both growth plate tissue and cultured chondrocytes express mRNA for EP1 and EP2.

**Figure 8:**
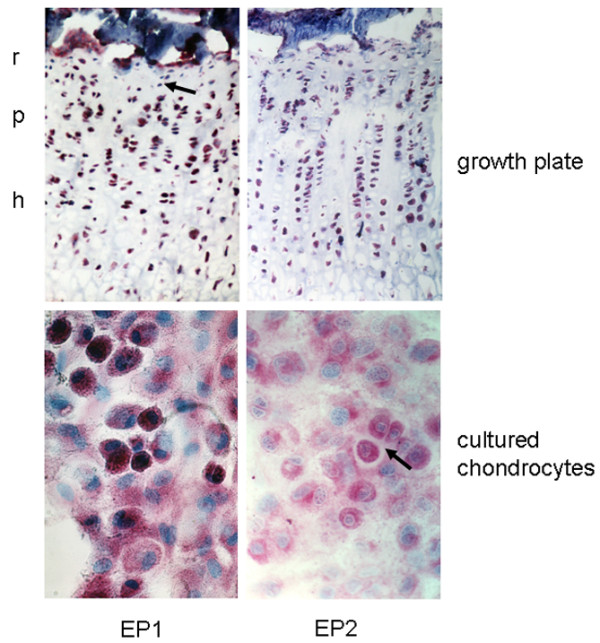
Immunohistochemical detection of EP1 and EP2 receptor in rat growth plates and in cultured chondrocytes. Protein expression of EP1 and EP2 receptor was studied using isoform-specific antibodies. The EP1 receptor showed strong expression in the proliferative and hypertrophic zone but marginal expression in the reserve zone, with some negative cells (marked by arrow). In contrast, the EP2 receptor was distributed throughout the whole growth plate. *In vitro *the EP1 and EP2 receptors were only expressed in subpopulations. EP1 showed strong positivity in chondrocytes organised in a cobblestone pattern and surrounded by matrix, whereas fibroblastic-shaped cells were only occasionally and moderately positive for EP1. The highest expression for EP2 could be demonstrated in dividing cells and polygonal cells embedded in matrix (marked by arrow). In fibroblastic cells, only minimal to slight positivity was found in a small number of cells. Magnification 200 × . r, reserve zone; p, proliferative zone; h, hypertrophic zone.

The expression of the different EP receptors was studied at the mRNA level by PCR and at the protein level by immunohistochemistry. The specificity of the antibodies used was assessed by omitting the first antibody and by preabsorbing with the corresponding peptide against which the antibody was generated. Under both conditions specific staining was absent (data not shown). Growth plate tissue as well as cultured chondrocytes showed expression of EP1 and EP2 receptor mRNA detected by reverse transcription-PCR (Figure 7). Regarding protein expression of the EP1 and EP2 receptors, the antibody against the EP2 receptor labelled all zones of the epiphyseal growth plate in a homogeneous manner. EP1 expression showed a different expression pattern, with strong expression in the proliferative and hypertrophic zone and only moderate expression in the reserve zone, occasionally with EP1 negative cells (Figure 8). In cultured chondrocytes, staining for EP1 was intense in confluent polygonal cells, which were organised in a cobblestone pattern and surrounded by matrix, whereas fibroblastic shaped cells were only occasionally positive. The EP2 receptor protein was expressed in distinct chondrocytes only. High expression was detected in dividing cells and polygonal chondrocytes embedded in matrix, whereas fibroblastic, and less differentiated chondrocytes showed only marginal staining in a small number of cells.

### Expression of EP3 and EP4 receptors

**Figure 9:**
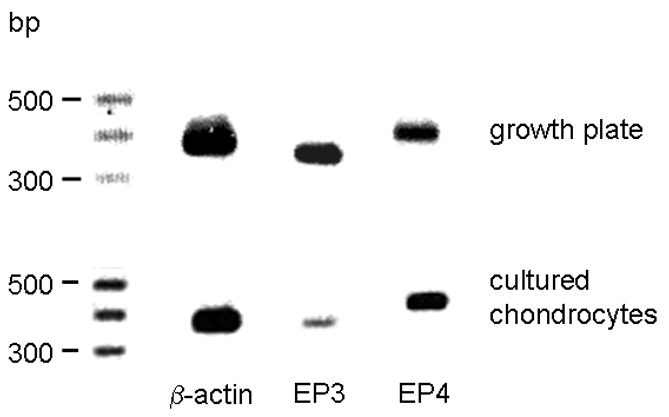
Expression of EP3 and EP4 receptor mRNA in rat growth plates and in cultured chondrocytes. Expression of mRNA for EP3 and EP4 receptor was analysed by RT-PCR. β-actin was used as positive control. Both growth plate tissue and cultured chondrocytes express mRNA for EP3 and EP4.

**Figure 10:**
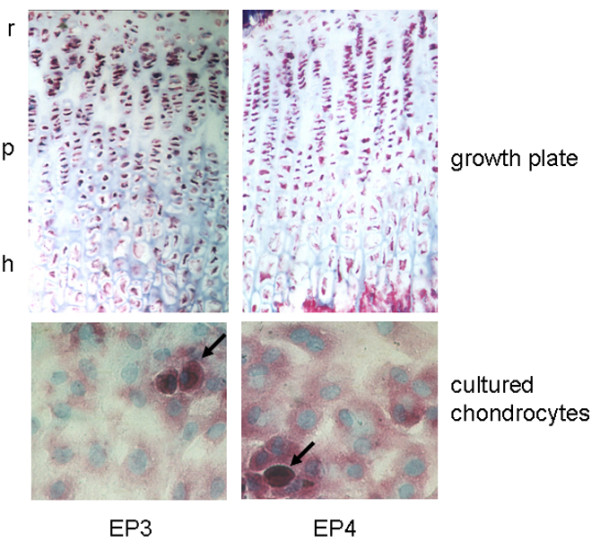
Immunohistochemical detection of EP3 and EP4 receptor proteins in rat growth plates and in cultured chondrocytes. Protein expression of EP3 and EP4 receptor was studied in growth plate tissue and cultured chondrocytes using isoform-specific antibodies. The EP3 and EP4 receptors were distributed throughout the whole growth plate. Cultured chondrocytes exhibited only weak reactivity towards the anti-EP antibodies. Only a minor subpopulation of cells showed strong staining for EP3 receptor and EP4 receptor. Magnification: 200 × . r, reserve zone; p, proliferative zone; h, hypertrophic zone.

Growth plate tissue as well as cultured chondrocytes showed expression of EP3 and EP4 receptor mRNA, although the amplification product for EP3 appeared to be less intense in the chondrocytes (Figure 9). In growth plates, EP3 and EP4 receptors were expressed in all layers (Figure 10). In cultured chondrocytes, a weak staining for both types of receptor was visible (Figure 9). Only distinct cells, which represent less than 10%, exhibited a strong reaction against the antibodies used.

## Discussion

The present study clearly demonstrates that growth plate chondrocytes are capable of secreting PGE_2_. The effects of PGE_2 _are mediated by G-protein-coupled receptors with different pathways of signal transduction. The present data show for the first time expression of COX-1 and COX-2, as well as EP1, EP2, EP3 and EP4, in the intact growth plate *in situ *in comparison with the expression in cultured growth plate chondrocytes. COX enzymes are expressed *in situ *in a characteristic spatial distribution: whereas COX-1 is homogenously expressed in all zones of the growth plate, COX-2 showed moderate expression in the reserve zone and strong expression in the other zones. Regarding EP receptor expression, EP1 expression *in situ *was mainly restricted to the proliferative and hypertrophic zone. Contrasting with this, EP2, EP3 and EP4 receptors *in situ *were homogeneously expressed by all chondrocytes, but *in vitro *by a subpopulation of cells only.

Collagen expression was analysed as a parameter of the phenotypic integrity of the chondrocytes and Col II and Col X are expressed in specific maturation states. In our system, the differentiation state of the majority of cells corresponded to cells in the proliferative layer, as shown previously [33]. This is confirmed not only by the proliferative activity but also by the production of Col II, and the lack of Col X, which is a specific marker of late hypertrophic chondrocytes [34]. Col I is not believed to be characteristically expressed in the growth plate and costochondral cartilage, but rather in the superficial layer of mandibular and articular cartilage [35]. Col I was also detectable in our cultured cells, which indicates the presence of 'de-differentiated' chondrocytes [28] in the absence of Col X expression.

PGE_2 _is produced by COX, of which two isoforms – COX-1 and COX-2 – exist. However, its protein expression has not been demonstrated previously in the growth plate, despite the fact that secreted prostanoids, which were generated by COX-1 and/or COX-2, were shown to modulate chondrocyte proliferation and function in *in vitro *systems. These results can only be extrapolated to the *in situ *situation if COX is expressed in the intact growth plate. Using polyclonal antibodies to COX-1 and COX-2, we were able to demonstrate COX-1 and COX-2 immunoreactivity in growth plate chondrocytes. Paralleling the *in situ *situation, both COX-1 and COX-2 mRNA as well as COX-1 and COX-2 protein were expressed in cultured chondrocytes. Concluding from the observed inhibitory effect of the COX-2 inhibitor SC-236, but not of the COX-1 inhibitor SC-560, on chondrocyte proliferation, we suggest that, at least for the cultured chondrocytes, COX-2 is the responsible enzyme driving PGE_2 _formation.

In our primary culture system, PGE_2 _stimulated DNA synthesis in a bell-shaped manner, the strongest effect being observed at concentrations that are higher than those physiologically found in the circulation [36]. These results are in accordance with studies by O'Keefe and colleagues [7] and Schwartz and colleagues [6], describing a growth-stimulatory effect of PGE_2 _at similar concentrations. We speculate, therefore, that secreted PGE_2 _could function as an autocrine/paracrine mediator of chondrocyte proliferation. From *in vitro *studies it is well known that PGE_2_may have different concentration-dependent effects on cell proliferation and matrix synthesis. This implies that local PGE_2 _concentrations in the various zones of the growth plate may differ. In fact, bovine chondrocytes isolated from the 'superficial zone' of the growth plate, that is, mainly reserve zone cells, were shown to produce less PGE_2 _than proliferating and early hypertrophic cells isolated from the 'deep zone' [37].

The proliferative action of PGE_2 _was mimicked by sulprostone, which was shown to selectively bind to EP1 and EP3 receptors [38] and only a minor stimulatory effect was provoked by misoprostole. Furthermore, a selective EP1 agonist provoked a similar proliferative effect in rat cultured chondrocytes compared to PGE_2 _and the growth-promoting effect of PGE_2 _could be completely blocked by a specific EP1 antagonist. We conclude that PGE_2 _mediates its proliferative effect primarily via the EP1 receptor. It has to be noted that a minor growth-promoting effect was also seen by the addition of EP2, EP3 and EP4 specific ligands. The minor growth-promoting effect observed with the EP3 agonist might be due to the presence of endogenously produced PGE_2_. EP3 receptor activation causes a decrease in intracellular cAMP levels. We speculate that in cultured chondrocytes, EP3 activation might promote an EP1 signalling pathway, triggered by endogenously formed PGE_2_, by ablation of cAMP, the opponent of the Ca^2+ ^signalling pathway. Alternatively, it has been shown that different splice variants do exist for the EP3 receptor, which in part may evoke a phosphatidyl-inositol response [18]. However, we can not exclude that different subpopulations within our cell culture system are regulated in a different way by PGE_2_, as we did not observe a homogenous expression of the different EP receptors in the cultured chondrocytes. Differences in responsiveness to PGE_2 _has, for example, also been reported for mouse chondroprogenitors and chondrocytes [39].

The second messenger of the EP1 receptor is free ionised intracellular calcium [40]. An increase of intracellular calcium was shown to be necessary for chondrocyte proliferation in response to the calciotropic hormones parathormone and 1,25(OH)_2_D_3 _[21,41]. The latter is thought to stimulate cell growth via generation of PGE_2 _[42]. To our knowledge, an increase of intracellular calcium in response to PGE_2 _has not been measured in growth plate chondrocytes. Contrasting with this hypothesis, PGE_2 _was found to have no effect on intracellular calcium in cultured articular bovine cartilage cells [43].

Corresponding to the proposed proliferative action of PGE_2 _via the EP1 receptor, this receptor could be demonstrated at the mRNA and protein levels not only *in vitro *but also *in situ*. In the intact growth plate we observed a strong EP1 receptor immunoreactivity in proliferative and hypertrophic chondrocytes, but not in reserve zone cells. This is in line with the proliferative effect of PGE_2 _mediated via the EP1 receptor. *In vitro*, EP1 was expressed in all cells, although the intensity varied. Because in our culture system proliferative cells represented the majority of chondrocytes, the ubiquitous expression of EP1 receptor *in vitro *was in contrast to the *in situ *situation. This discrepancy indicates that extrapolation of the *in vitro *data to the *in situ *situation should be done with caution.

In addition, the EP2 receptor also showed a different expression pattern *in situ *and *in vitro*. The EP2 receptor was not uniformly detectable *in vitro*, although *in situ *all cells were positive. The highest expression was observed in dividing cells. It can be concluded from our data that EP2 receptor signalling also contributes to cell growth. The inhomogenous expression of EP2 in cultured chondrocytes may explain the lower proliferative effect achieved by the specific EP2 agonist. EP2 receptor expression has also been described in cultured articular chondrocytes [43] and fourth passage reserve zone cells [44]. In the latter, PGE_2 _stimulated intracellular cAMP, which resulted in increased matrix synthesis. In a chondrocyte cell line, established from articular cartilage of p53^-/- ^mice, the EP2 receptor was identified as the major PGE_2 _receptor [45]. In this cell line, EP2 agonists evoked cAMP generation and promoted cell growth. In articular chondrocytes, PGE_2 _probably mediates its proliferative effect primarily via the EP2 receptor whereas in growth plate chondrocytes the EP1 receptor is dominant for PGE_2_-dependent growth. EP2 and EP4 receptors may also be involved in chondrogenesis [39]. In limb bud mesenchymal cells, all four types of EP receptor are expressed and EP2 and EP4 receptor activation of cAMP metabolism was suggested to drive mesenchymal stem cells to chondrogenesis. We observed a weak expression of the EP4 receptor in our cultured chondrocytes. Most likely, EP receptors, and especially the EP4 type, are expressed depending on the cell differentiation state in culture. By contrast, in the growth plate tissue of the rat we observed EP4 expression in all layers. In a recent study, Miyamoto and colleagues [20] showed that the EP2 receptor promotes differentiation and synthesis of Col II and proteoglycans in cultured bovine growth plate cells. This effect was dependent on co-stimulation of the EP4 receptor; however, in rat, the EP4 receptor was not detected, at least in fourth passage chondrocytes [46]. In view of these results, a role for the EP2 receptor in chondrocyte differentiation can be hypothesised. The differentiation-dependent expression of EP receptors might explain the contradictory results obtained in studies investigating the effects of PGE_2_. This indicates the crucial role played by species and culture conditions used in the various *in vitro *systems. According to our *in vivo *data, all types of EP receptors appeared to be expressed. Taking into account that the different EP receptors are coupled to different intracellular signalling pathways, we expect that other mechanisms, such as receptor activation, modulation of ligand affinity or selective access of PGE_2 _to the necessary receptor type, are involved in ensuring a coordinated action of PGE_2 _in growth plate physiology.

## Conclusion

Cultured growth plate chondrocytes synthesized PGE_2_. Exogenous PGE_2 _stimulation had a proliferating-inducing effect in a dose-dependent manner on cultured growth plate chondrocytes via the EP1 receptor, which could be mimicked by EP agonists such as sulprostone and ONO-D1-004. The proliferating effects could be blocked by the EP1 antagonist ONO-8713.

Further analyses of the physiological and pathophysiological roles of EP1 and EP2, especially in chronic inflammatory disorders, are needed. From a therapeutic point of view, the long term effects of COX inhibitors and EP antagonists with respect to the integrity of the growth plate in the paediatric population is of special interest. Growth plate chondrocytes express COX-1, COX-2 and EP1, EP2, EP3, and EP4 *in situ *and *in vitro *with markedly different expression patterns. Therefore, the extrapolation from *in vitr*o data to the *in situ *situation and the interpretation regarding physiological processes must be done with caution.

With respect to the possibilities for cartilage regeneration in the context of tissue engineering of bone and cartilage, the present data open interesting new aspects for optimising the seeding of scaffolds via stimulation of cell proliferation by PGE_2 _or EP1 ligands; at present, this is under investigation. The analysis of arachidonic metabolites in the growth plate *in vitro *and *in situ *presents a wide scope for further investigations with pathophysiological, therapeutic and regenerative end points.

## Abbreviations

Col = collagen; COX = cyclooxygenase; DMEM = Dulbecco's modified Eagle's medium; EP = prostaglandin E receptor; FCS = fetal calf serum; PGE_2 _= prostaglandin E_2_.

## Competing interests

The authors declare that they have no competing interests.

## Authors' contributions

CB and PN made substantial contributions to the conception and design of experiments, data acquisition, analysis and interpretation; they were also involved in manuscript drafting and revising and contributed equally to this work. RMN performed statistical analysis, made substantial contributions to analysis and interpretation of data and was involved in drafting the manuscript. CJK was involved in data interpretation, drafting the manuscript and revised it critically for the physiological and pathophysiological impact of the data. GK made substantial contributions to the conception and design of the experiments as well as to interpretation of data and was involved in drafting the manuscript. All authors read and approved the final manuscript.

## References

[B1] Klein DC Raisz LG Prostaglandins: stimulation of bone resorption in tissue culture Endocrinology 1970 86 1436 1440 4315103 10.1210/endo-86-6-1436

[B2] Flanagan AM Chambers TJ Stimulation of bone nodule formation *in vitro* by prostaglandins E1 and E2 Endocrinology 1992 130 443 448 10.1210/en.130.1.443 1309342

[B3] Weinreb M Rutledge SJ Rodan GA Systemic administration of an anabolic dose of prostaglandin E(2) induces early-response genes in rat bones Bone 1997 20 347 353 10.1016/S8756-3282(97)00011-2 9108355

[B4] Igarashi K Hirafuji M Adachi H Shinoda H Mitani H Role of endogenous PGE2 in osteoblastic functions of a clonal osteoblast-like cell, MC3T3-E1 Prostaglandins Leukot Essent Fatty Acids 1994 50 169 172 10.1016/0952-3278(94)90140-6 8022850

[B5] Lowe GN Fu YH McDougall S Polendo R Williams A Benya PD Hahn TJ Effects of prostaglandins on deoxyribonucleic acid and aggrecan synthesis in the RCJ 3.1C5.18 chondrocyte cell line: role of second messengers Endocrinology 1996 137 2208 2216 10.1210/en.137.6.2208 8641167

[B6] Schwartz Z Gilley RM Sylvia VL Dean DD Boyan BD The effect of prostaglandin E2 on costochondral chondrocyte differentiation is mediated by cyclic adenosine 3',5'-monophosphate and protein kinase C Endocrinology 1998 139 1825 1834 10.1210/en.139.4.1825 9528968

[B7] O'Keefe RJ Crabb ID Puzas JE Rosier RN Influence of prostaglandins on DNA and matrix synthesis in growth plate chondrocytes J Bone Miner Res 1992 7 397 404 1319104 10.1002/jbmr.5650070407

[B8] Ueda K Saito A Nakano H Aoshima M Yokota M Muraoka R Iwaya T Cortical hyperostosis following long-term administration of prostaglandin E1 in infants with cyanotic congenital heart disease J Pediatr 1980 97 834 836 10.1016/S0022-3476(80)80282-4 7000997

[B9] Suponitzky I Weinreb M Differential effects of systemic prostaglandin E2 on bone mass in rat long bones and calvariae J Endocrinol 1998 156 51 57 10.1677/joe.0.1560051 9496233

[B10] Marks SC Jr Miller S Local infusion of prostaglandin E1 stimulates mandibular bone formation *in vivo* J Oral Pathol 1988 17 500 505 10.1111/j.1600-0714.1988.tb01324.x 3150437

[B11] Yang RS Liu TK Lin-Shiau SY Increased bone growth by local prostaglandin E2 in rats Calcif Tissue Int 1993 52 57 61 10.1007/BF00675627 8453506

[B12] Vane JR Bakhle YS Botting RM Cyclooxygenases 1 and 2 Annu Rev Pharmacol Toxicol 1998 38 97 120 10.1146/annurev.pharmtox.38.1.97 9597150

[B13] Sylvia VL Del Toro F Dean DD Hardin RR Schwartz Z Boyan BD Effects of 1alpha,25-(OH)(2)D(3) on rat growth zone chondrocytes are mediated via cyclooxygenase-1 and phospholipase A(2) J Cell Biochem 2001 81 32 45 10.1002/jcb.1072 11455568

[B14] Simon AM Manigrasso MB O'Connor JP Cyclo-oxygenase 2 function is essential for bone fracture healing J Bone Miner Res 2002 17 963 976 10.1359/jbmr.2002.17.6.963 12054171

[B15] Sakai T Kambe F Mitsuyama H Ishiguro N Kurokouchi K Takigawa M Iwata H Seo H Tumor necrosis factor alpha induces expression of genes for matrix degradation in human chondrocyte-like HCS-2/8 cells through activation of NF-kappaB: abrogation of the tumor necrosis factor alpha effect by proteasome inhibitors J Bone Miner Res 2001 16 1272 1280 10.1359/jbmr.2001.16.7.1272 11450703

[B16] Abulencia JP Gaspard R Healy ZR Gaarde WA Quackenbush J Konstantopoulos K Shear-induced cyclooxygenase-2 via a JNK2/c-Jun-dependent pathway regulates prostaglandin receptor expression in chondrocytic cells J Biol Chem 2003 278 28388 28394 10.1074/jbc.M301378200 12743126

[B17] Siegle I Klein T Backman JT Saal JG Nüsing RM Fritz P Expression of cyclooxygenase 1 and cyclooxygenase 2 in human synovial tissue: Differential elevation of cyclooxygenase 2 in inflammatory joint diseases Arthritis Rheum 1998 41 122 129 10.1002/1529-0131(199801)41:1<122::AID-ART15>3.0.CO;2-8 9433877

[B18] Narumiya S Sugimoto Y Ushikubi F Prostanoid receptors: structures, properties, and functions Physiol Rev 1999 79 1193 1226 10508233 10.1152/physrev.1999.79.4.1193

[B19] Yoshida K Oida H Kobayashi T Maruyama T Tanaka M Katayama T Yamaguchi K Segi E Tsuboyama T Matsushita M Stimulation of bone formation and prevention of bone loss by prostaglandin E EP4 receptor activation Proc Natl Acad Sci USA 2002 99 4580 4585 10.1073/pnas.062053399 11917107 PMC123690

[B20] Miyamoto M Ito H Mukai S Kobayashi T Yamamoto H Kobayashi M Maruyama T Akiyama H Nakamura T Simultaneous stimulation of EP2 and EP4 is essential to the effect of prostaglandin E2 in chondrocyte differentiation Osteoarthritis Cartilage 2003 11 644 652 10.1016/S1063-4584(03)00118-3 12954235

[B21] Klaus G von Eichel B May T Hügel U Mayer H Ritz E Mehls O Synergistic effects of parathyroid hormone and 1,25-dihydroxyvitamin D3 on proliferation and vitamin D receptor expression of rat growth cartilage cells Endocrinology 1994 135 1307 1315 10.1210/en.135.4.1307 7523093

[B22] Erdmann S Müller W Bahrami S Vornehm SI Mayer H Bruckner P von der Mark K Burkhardt H Differential effects of parathyroid hormone fragments on collagen gene expression in chondrocytes J Cell Biol 1996 135 1179 1191 10.1083/jcb.135.4.1179 8922395 PMC2133384

[B23] Kömhoff M Gröne HJ Klein T Seyberth HW Nüsing RM Localization of cyclooxygenase-1 and -2 in adult and fetal human kidney: implication for renal function Am J Physiol 1997 272 F460 468 9140046 10.1152/ajprenal.1997.272.4.F460

[B24] Morath R Klein T Seyberth HW Nüsing RM Immunolocalization of the four prostaglandin E2 receptor proteins EP1, EP2, EP3, and EP4 in human kidney J Am Soc Nephrol 1999 10 1851 1860 10477136 10.1681/ASN.V1091851

[B25] Kawamori T Uchiya N Kitamura T Ohuchida S Yamamoto H Maruyama T Sugimura T Wakabayashi K Evaluation of a selective prostaglandin E receptor EP1 antagonist for potential properties in colon carcinogenesis Anticancer Res 2001 21 3865 3869 11911260

[B26] Kiriyama M Ushikubi F Kobayashi T Hirata M Sugimoto Y Narumiya S Ligand binding specificities of the eight types and subtypes of the mouse prostanoid receptors expressed in Chinese hamster ovary cells Br J Pharmacol 1997 122 217 224 10.1038/sj.bjp.0701367 9313928 PMC1564924

[B27] Suzawa T Miyaura C Inada M Maruyama T Sugimoto Y Ushikubi F Ichikawa A Narumiya S Suda T The role of prostaglandin E receptor subtypes (EP1, EP2, EP3, and EP4) in bone resorption: an analysis using specific agonists for the respective EPs Endocrinology 2000 141 1554 1559 10.1210/en.141.4.1554 10746663

[B28] Benya PD Shaffer JD Dedifferentiated chondrocytes reexpress the differentiated collagen phenotype when cultured in agarose gels Cell 1982 30 215 224 10.1016/0092-8674(82)90027-7 7127471

[B29] Bohme K Conscience-Egli M Tschan T Winterhalter KH Bruckner P Induction of proliferation or hypertrophy of chondrocytes in serum-free culture: the role of insulin-like growth factor-I, insulin, or thyroxine J Cell Biol 1992 116 1035 1042 10.1083/jcb.116.4.1035 1734018 PMC2289336

[B30] Cordell JL Falini B Erber WN Ghosh AK Abdulaziz Z MacDonald S Pulford KA Stein H Mason DY Immunoenzymatic labeling of monoclonal antibodies using immune complexes of alkaline phosphatase and monoclonal anti-alkaline phosphatase (APAAP complexes) J Histochem Cytochem 1984 32 219 229 6198355 10.1177/32.2.6198355

[B31] Bittinger F Brochhausen C Kohler H Lehr HA Otto M Skarke C Walgenbach S Kirkpatrick CJ Differential expression of cell adhesion molecules in inflamed appendix: correlation with clinical stage J Pathol 1998 186 422 428 10.1002/(SICI)1096-9896(199812)186:4<422::AID-PATH209>3.0.CO;2-7 10209493

[B32] Schweer H Watzer B Seyberth HW Determination of seven prostanoids in 1 ml of urine by gas chromatography-negative ion chemical ionization triple stage quadrupole mass spectrometry J Chromatogr 1994 652 221 227 8006107 10.1016/0378-4347(93)e0408-i

[B33] Balmain N von Eichel B Toury R Belquasmi F Hauchecorne M Klaus G Mehls O Ritz E Calbindin-D28K and -D9K and 1,25(OH)2 vitamin D3 receptor immunolocalization and mineralization induction in long-term primary cultures of rat epiphyseal chondrocytes Bone 1995 17 37 45 10.1016/8756-3282(95)00132-W 7577156

[B34] O'Keefe RJ Puzas JE Loveys L Hicks DG Rosier RN Analysis of type II and type X collagen synthesis in cultured growth plate chondrocytes by in situ hybridization: rapid induction of type X collagen in culture J Bone Miner Res 1994 9 1713 1722 7863822 10.1002/jbmr.5650091107

[B35] Fukunaga T Yamashiro T Oya S Takeshita N Takigawa M Takano-Yamamoto T Connective tissue growth factor mRNA expression pattern in cartilages is associated with their type I collagen expression Bone 2003 33 911 918 10.1016/j.bone.2003.07.010 14678850

[B36] Schweer H Kammer J Kühl PG Seyberth HW Determination of peripheral plasma prostanoid concentration: an unreliable index of "*in vivo*" prostanoid activity Eur J Clin Pharmacol 1986 31 303 305 10.1007/BF00981128 3466793

[B37] Chowdhury TT Bader DL Lee DA Dynamic compression counteracts IL-1 beta-induced release of nitric oxide and PGE2 by superficial zone chondrocytes cultured in agarose constructs Osteoarthritis Cartilage 2003 11 688 696 10.1016/S1063-4584(03)00149-3 12954240

[B38] Watabe A Sugimoto Y Honda A Irie A Namba T Negishi M Ito S Narumiya S Ichikawa A Cloning and expression of cDNA for a mouse EP1 subtype of prostaglandin E receptor J Biol Chem 1993 268 20175 20178 7690750

[B39] Clark CA Schwarz EM Zhang X Ziran NM Drissi H O'Keefe RJ Zuscik MJ Differential regulation of EP receptor isoforms during chondrogenesis and chondrocyte maturation Biochem Biophys Res Commun 2005 328 764 776 10.1016/j.bbrc.2004.11.074 15694412

[B40] Funk CD Furci L FitzGerald GA Grygorczyk R Rochette C Bayne MA Abramovitz M Adam M Metters KM Cloning and expression of a cDNA for the human prostaglandin E receptor EP1 subtype J Biol Chem 1993 268 26767 26772 8253813

[B41] Klaus G König B Hügel U Ritz E Mehls O Intermittent and continuous exposure to 1,25(OH)2D3 have different effects on growth plate chondrocytes *in vitro* Kidney Int 1993 44 708 715 8258948 10.1038/ki.1993.304

[B42] Schwartz Z Gilley RM Sylvia VL Dean DD Boyan BD Prostaglandins mediate the effects of 1,25-(OH)2D3 and 24,25-(OH)2D3 on growth plate chondrocytes in a metabolite-specific and cell maturation-dependent manner Bone 1999 24 475 484 10.1016/S8756-3282(99)00014-9 10321907

[B43] de Brum-Fernandes AJ Morisset S Bkaily G Patry C Characterization of the PGE2 receptor subtype in bovine chondrocytes in culture Br J Pharmacol 1996 118 1597 1604 8842420 10.1111/j.1476-5381.1996.tb15580.x PMC1909846

[B44] Del Toro F Jr Sylvia VL Schubkegel SR Campos R Dean DD Boyan BD Schwartz Z Characterization of prostaglandin E(2) receptors and their role in 24,25-(OH)(2)D(3)-mediated effects on resting zone chondrocytes J Cell Physiol 2000 182 196 208 10.1002/(SICI)1097-4652(200002)182:2<196::AID-JCP8>3.0.CO;2-E 10623883

[B45] Aoyama T Liang B Okamoto T Matsusaki T Nishijo K Ishibe T Yasura K Nagayama S Nakayama T Nakamura T PGE2 signal through EP2 promotes the growth of articular chondrocytes J Bone Miner Res 2005 20 377 389 10.1359/JBMR.041122 15746982

[B46] Sylvia VL Del Toro F Jr Hardin RR Dean DD Boyan BD Schwartz Z Characterization of PGE(2) receptors (EP) and their role as mediators of 1alpha,25-(OH)(2)D(3) effects on growth zone chondrocytes J Steroid Biochem Mol Biol 2001 78 261 274 10.1016/S0960-0760(01)00099-1 11595507

